# Preclinical Assessment of wt GNE Gene Plasmid for Management of Hereditary Inclusion Body Myopathy 2 (HIBM2)

**DOI:** 10.4137/grsb.s728

**Published:** 2008-06-20

**Authors:** Chris Jay, Gregory Nemunaitis, John Nemunaitis, Neil Senzer, Stephan Hinderlich, Daniel Darvish, Julie Ogden, John Eager, Alex Tong, Phillip B Maples

**Affiliations:** 1 Gradalis, Inc., Dallas, TX; 2 Mary Crowley Cancer Research Centers, Dallas, TX; 3 University of Applied Sciences Berlin, Department of Life Sciences and Technology, Germany; 4 HIBM Research Group, Encino, CA; 5 MetroHealth Medical Center, Cleveland, OH; 6 Sammons Cancer Center, Baylor University Medical Center, Dallas, TX

## Abstract

Hereditary Inclusion Body Myopathy (HIBM2) is a chronic progressive skeletal muscle wasting disorder which generally leads to complete disability before the age of 50 years. There is currently no effective therapeutic treatment for HIBM2. Development of this disease is related to expression in family members of an autosomal recessive mutation of the GNE gene, which encodes the bifunctional enzyme UDP-GlcNAc 2-epimerase/ManNAc kinase (GNE/MNK). This is the rate limiting bifunctional enzyme that catalyzes the first 2 steps of sialic acid biosynthesis. Decreased sialic acid production, consequently leads to decreased sialyation of a variety of glycoproteins including the critical muscle protein alpha-dystroglycan (α-DG). This in turn severely cripples muscle function and leads to the onset of the syndrome. We hypothesize that replacing the mutated GNE gene with the wildtype gene may restore functional capacity of GNE/MNK and therefore production of sialic acid, allowing for improvement in muscle function and/or delay in rate of muscle deterioration. We have constructed three GNE gene/CMV promoter plasmids (encoding the wildtype, HIBM2, and Sialuria forms of GNE) and demonstrated enhanced GNE gene activity following delivery to GNE-deficient CHO-Lec3 cells. GNE/MNK enzyme function was significantly increased and subsequent induction of sialic acid production was demonstrated after transfection into Lec3 cells with the wild type or R266Q mutant GNE vector. These data form the foundation for future preclinical and clinical studies for GNE gene transfer to treat HIBM2 patients.

## Introduction

Hereditary Inclusion Body Myopathy 2 (HIBM2) is an autosomal recessive muscle wasting syndrome with its onset between the ages of 20 and 40 years of age progressing to severe incapacitation within 5 to 15 years (Argov and Yarom, 1984). Etiology for this disorder is based on the presence of mutations of the GNE gene. Worldwide an estimated 271 cases are reported, 159 in the Middle East (Iranian-Jewish heritage) which bear the M712T founder mutation and 42 are reported to have the V572L founder mutation, identified in the Japanese population (see http://hibm.org/hrg/pages/research-services/gne/ibm2-mutations.php). GNE is a highly conserved gene identified as 1 of 40 genes exclusively shared by vertebrates and bacteria (Salzberg, White et al. 2001). It encodes the bifunctional enzyme uridine diphosphate-N-acetylglucosamine 2—epimerase/N-acetylmannosamine kinase (GNE/MNK) which catalyzes the first two sequential sialic acid pathway reactions critical to sialic acid synthesis, and HIBM2 mutations are distributed over the whole enzyme (Argov, Eisenberg et al. 2003; Krause, Schlotter-Weigel et al. 2003; Broccolini, Ricci et al. 2004; Huizing, Rakocevic et al. 2004; Saito, Tomimitsu et al. 2004).

Sialic acid is the only monosaccharide of glycoconjugates that bears a net negative charge (Alberts, Bray et al. c1994). This charged sugar provides the terminal carbohydrate on a variety of N-linked and O-linked glycoproteins that mediate cell-cell and protein-protein interactions. The sialic acid modifications of cell surface glycoproteins are crucial for cell adhesion and signal transduction and may result in muscle fiber degeneration (Huizing, Hermos et al. 2002; Vasconcelos, Raju et al. 2002). Although sialic acid dysregulation is likely to significantly contribute to disease pathogenesis, recent assessments of myoblast cellular sialylation patterns suggest the possible role of other GNE related contributing mechanisms, such as GM3 and GD3 synthase regulation and impaired apoptosis signalling (Hinderlich, Salama et al. 2004; Salama, Hinderlich et al. 2005; Wang, Sun et al. 2006; Amsili, Shlomai et al. 2007). Additionally, recent developments in the field of GNE research has implicated the bifunctional enzyme to also play a role in functions other than sialic acid production. These functions include cell proliferation, apoptosis, regulatory gene expression and modulatory sialytransferases (Wang, Sun et al. 2006; Amsili, Shlomai et al. 2007).

There is no current effective treatment for HIBM2, unlike the related disorders of inflammatory myopathies (polymyositis and dermatomyositis) that respond somewhat to corticosteroids, plasmaphoresis/filtration (removal of immunogenic proteins) or other immunosuppressive therapies (Dalakas, 1991; Oldfors and Lindberg, 1999). In an initial double-blind, placebo-controlled trial involving 19 individuals utilizing immunoglobulins (IVIg) as treatment for sporadic IBM, no statistically significant beneficial effects were observed (Dalakas, Sonies et al. 1997). Similar results were demonstrated in a more recent study involving 4 patients with HIBM2 treated with IVIg containing 8 μmoles sialic acid per gram (Sparks, Rakocevic et al. 2007). Even though some improvement in muscle function with variable and transient clinical improvement with daily activities was observed, no objective evidence of improved muscle sialylation at post treatment muscle biopsy was demonstrated (Sparks, Rakocevic et al. 2007). A third study involving 36 patients with sporadic IBM in which IVIg was combined with prednisone also demonstrated no effectiveness in improving muscle function (Dalakas, Koffman et al. 2001).

We hypothesize a novel therapeutic opportunity for HIBM2 using a newly constructed wildtype GNE gene plasmid thereby addressing both sialic acid based and alternative GNE-based mechanisms of cellular dysfunction and have demonstrated GNE transgene expression along with sialic production in GNE defective CHO-Lec 3 cells.

## Materials and Methods

### GNE cloning

Parental vectors containing the GNE cDNA were provided by Daniel Darvish (HIBM Research Group; Encino, CA) and included pGNE-NB8 (wild type), pGNE-MB18 (M712T mutant), and pGNE-R266Q (R266Q mutant). The destination vector, pUMVC3, was purchased from Aldevron (Fargo, ND). The subcloning vector, pDrive (Qiagen; Valencia, CA), was used to shuttle the R266Q mutant from the parent vector to the destination vector.

**Figure 1:**
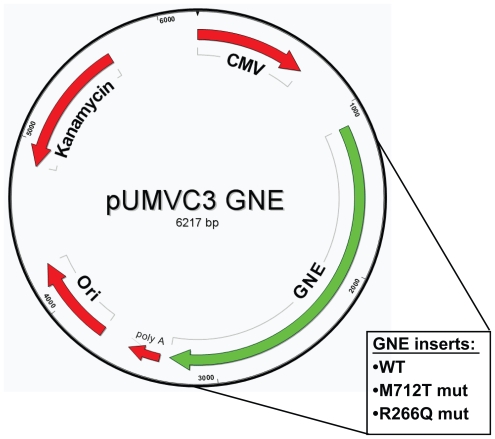
pUMVC3-GNE expression vector.

Wild type and M712T GNE was cloned from the parent vector into pUMVC3 via Eco RI restriction digest, gel purification, and T4 ligation. The R266Q mutant GNE was cloned from the parent vector into pDrive via Hind III + Xba I digest and then moved to pUMVC3 via Sal I + Xba I. (Fig. 1) All pUMVC3-GNE clones were sequenced by Seqwright (Houston, TX) with the following primers: GNE-F1, 5′-*TGTGAGG ACCATGATCGCATCCTT*-3′; GNE-F2, 5′-*ACCTCCGAGTTGCAATAGTCAGCA*-3′; GNE-R1, 5′-*AATCAGGCCCATCCAGAGACACAA*-3′; GNE-R2, 5′-*TTCCAATCTGACG TGTTCCCAGGT*-3′; UMVC-F, 5′-*CGCCACCAGACATAATAGCT-GACA*-3′; and UMVC-R, 5′-*TAGCCAGA AGTCAGATGCTCAAGG*-3′. Positive pUMVC3-GNE clones were grown overnight in 175mls LB broth + 50 ug/ml Kan and 150 mls culture was used for the Qiagen (Valencia, CA) HiSpeed Plasmid Maxi kit according to the manufacturer protocols.

### Cell culture

GNE deficient CHO-Lec3 cells were provided by Pamela Stanley at Albert Einstein College of Medicine (Hong and Stanley, 2003). The cells were grown at 37 °C in 5% CO_2_ in alpha-MEM media supplemented with 4mM L-glutamine and 10% heat inactivated, Fetal Bovine Serum. Cells for transient transfections were plated at 1 × 10^6 cells per well in 6-well plates and grown overnight. Lec3 cells were weaned to reduced serum conditions by reducing the FBS by 2.5% per passage.

### Transient transfections

Lec3 cells were transfected for 6 hours with DNA: lipid complex per well in OptiMEM (Invitrogen; Carlsbad CA), then the media was changed to normal alpha-MEM growth media and the cells were cultured overnight. DNA:lipid complexes were formed by mixing 4 ug DNA + 10 ul Lipofectamine 2000 (Invitrogen) according to the manufacturers protocol. Twenty-four hours post transfection, cells were harvested by trypsin digest and washed once with PBS before subsequent western blot or enzyme/sugar assays.

### mRNA quantitation

Total RNA was extracted from 1.5 million transfected CHO-Lec3 cells using the RNeasy kit according to the manufacturers instructions (Qiagen, Valencia, CA). The purified RNA was quantified by 260/280 ratio using the NanoDrop1000 spectrophotometer (NanoDrop, Wilmington, DE). Five hundred nanograms of total RNA was converted to cDNA using oligo dT primers and the TaqMan reverse transcription kit (ABI, Foster City, CA). Using the Sybr Green PCR master mix (ABI, Foster City, CA) along with 25 ng cDNA and 0.2 pM primers (GNE-F3 = 5′-cggaagaagggcatt-gagcatc-3′ and GNE-R3 = 5′-tttgtcttgggtgtcag-catcc-3′), 25 ul PCR reactions were compared against serial dilutions of a known concentration of pUMVC3-GNE-wt DNA. The Sybr Green fluorescence was detected using the iQ5 real time PCR detection system (BioRad, Hercules, CA) and the PCR conditions: 95 °C–10 min to activate the enzyme and (95 °C–15 sec and 58 °C–60 sec) × 45 cycles to amplify the product. Fifteen microliters of the PCR reaction was run on a 4% pre-cast agarose E-gel (Invitrogen, Carlsbad, CA) and the image was captured using the G-box chemiluminescence detection system (Frederick, MD).

### Western blot

Approximately 5 × 10^5 cells were used for western blot analysis. Cell pellets were lysed using 20 ul Cell lytic (Sigma; St. Louis, MO) plus 1% protease inhibitors. The cell debris was spun down at maximum speed for 5 minutes and the supernatant was mixed 1:1 with Laemmli buffer (BioRad; Hercules, CA) containing 5% β-ME. Protein samples were separated by polyacrylamide electrophoresis at 100V for 2 hours on 10% denaturing gels, followed by transfer to PVDF membrane using 100 volts for 2 hours. The membranes were probed for GNE and GapDH using chicken anti-GNE (1:10,000 dilution) and mouse anti-GapDH (1:50,000 dilution) overnight. Primary antibodies were detected using HRP-labeled secondary antibodies and they were visualized using the West Dura detection reagent (Pierce; Rockford, IL) and the G-box chemiluminescence camera (Syngene; Frederick, MD).

### Sialic acid quantitation

Approximately 4 × 10^6 cells were used for the quantification of membrane-bound sialic acid by the thiobarbituric acid method. Cells were resuspended in water and lysed by passage through a 25 gauge needle 20 times and centrifuged. The supernatant was used for Bradford protein estimation and the remaining pellet was resuspended in 100 μl 2 M acetic acid and incubated 1 h at 80 °C to release glycoconjugate-bound sialic acids. 137 μl of periodic acid solution (2.5 mg/ml in 57 mM H_2_SO_4_) were added and incubated for 15 min at 37 °C. Then 50 μl of sodium arsenite solution (25 mg/ml in 0.5 M HCl) were added and the tubes were shaken vigorously to ensure complete elimination of the yellow-brown color. After this step 100 μl of 2-thiobarbituric acid solution (71 mg/ml adjusted to pH 9.0 with NaOH) were added and the samples were heated to 100 °C for 7.5 min. The solution was extracted with 1 ml of butanol/5% 12 M HCl and the phases were separated by centrifugation. The absorbance of the organic phase was measured at 549 nm. The amount of sialic acids was given as nmol sialic acid/mg of protein.

### Kinase and epimerase activity

UDP-GlcNAc 2-epimerase activity was determined by a colorimetric assay (Blume, Chen et al. 2002). It contained 45 mM Na_2_HPO_4_, pH 7.5, 10 mM MgCl_2_, 1 mM UDP-GlcNAc and variable amounts of protein in a final volume of 200 μl. The reaction was performed at 37 °C for 30 min and stopped by boiling for 1 min. The released Man-NAc was detected by the Morgan-Elson method (Reissig, Storminger et al. 1955). In brief, 150 μl of sample were mixed with 30 μl of 0.8 M H_2_BO_3_, pH 9.1, and boiled for 3 min. Then 800 μl of DMAB solution (1% (w/v) 4-dimethylamino benzaldehyde in acetic acid/1.25% 10 N HCl) was added and incubated at 37 °C for 30 min. The absorbance was read at 578 nm.

ManNAc kinase activity was measured by a radiometric assay (Hinderlich, Stasche et al. 1997). It contained 60 mM Tris/HCl, pH 8.1, 10 mM MgCl_2_, 5 mM ManNAc, 50 nCi [14C]ManNAc, 10 mM ATP, and variable amounts of protein in a final volume of 200 μl. The reaction was performed at 37 °C for 30 min and stopped by addition of 300 μl of ethanol. Radiolabeled compounds were separated by paper chromatography and radioactivity was determined by liquid scintillation counting.

### Statistical analysis

Independent experiments for enzyme activity and sialic acid expression were performed. The student’s t-test was used to determine p-values for each treated group, relative to the untreated sample. One way analysis of variance (ANOVA), followed by Duncan’s multiple comparisons test when appropriate, was used to determine p values for each treated group, relative to each other.

## Results

### Creating GNE clones

GNE cDNA clones tested included a human wild type cDNA and two human mutant cDNAs. The mutants included the M712T GNE deficient clone and the R266Q sialuria clone. Sialuria is a human disease caused by point mutations in the CMP-sialic acid binding site of GNE, leading to a loss of feed-back inhibition and mass production of sialic acids. GNE cDNAs were subcloned from their original vectors to the expression vector, pUMVC3 by restriction digest cloning. Clones were screened by directional restriction enzyme digest to confirm the GNE insert was in the correct orientation. (data not shown) Positive clones were sequenced in both orientations to confirm no mutations occurred during the cloning process. The resulting chromatograms were compared against the GNE sequence from GenBank (accession # NM_005467) and the wild type had no mutations, whilst the M712T and R266Q clones contained only the expected point mutations (Sup Fig. 1). Positive pUMVC3-GNE clones were scaled up by maxi prep plasmid purification and sequenced again to confirm no mutations occurred (data not shown). These DNA stocks were used for all subsequent experiments.

### Gene protein expression

**Figure 2:**
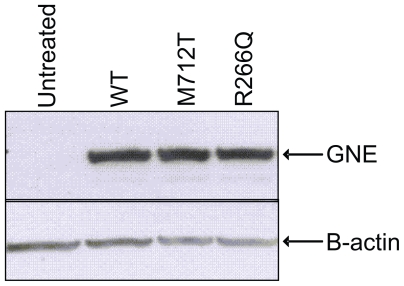
GNE expression in CHO-Lec3 cells grown in 10% serum. Lane 1: untreated Lec3 cells. Lane 2: wt GNE. Lane 3: M712T GNE. Lane 4: R266Q GNE.

**Figure 3:**
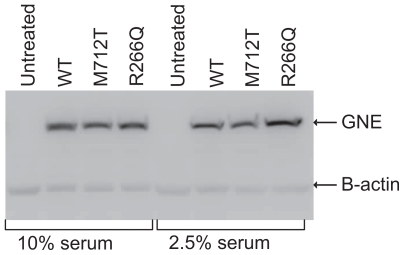
GNE expression in CHO-Lec3 cell lines. Lanes 1–4: CHO-Lec3 cells grown in 10% FBS. Lanes 5–8: CHO-Lec3 cells grown in 2.5% FBS. Lanes 1 and 5: Untreated Lec3 cells. Lanes 2 and 6: wt GNE. Lanes 3 and 7: M712T GNE. Lanes 4 and 8: R266Q GNE.

Plasmid UMVC3-GNE DNA was transiently transfected into CHO-Lec3 cells and grown in 10% serum for 24 hours, and then the cells were harvested and analyzed for recombinant GNE expression. The GNE western blot illustrates that the untreated Lec3 cells do not express GNE and CHO-Lec3 cells transfected with different pUMVC3 clones express high levels of recombinant GNE (Fig. 2). The expression level is relatively equivalent, regardless of GNE isoform. In a second experiment recombinant GNE was expressed following transfection of CHO-Lec3 cells grown in 10% or 2.5% fetal bovine serum (FBS), due to the ability of CHO cells to incorporate sialic acids from the culture media (see sialic acid quantification below). Again, GNE protein expression was relatively equivalent, regardless of GNE isoform and the concentration of FBS (Fig. 3).

### Wt-GNE mRNA quantitation

**Figure 4:**
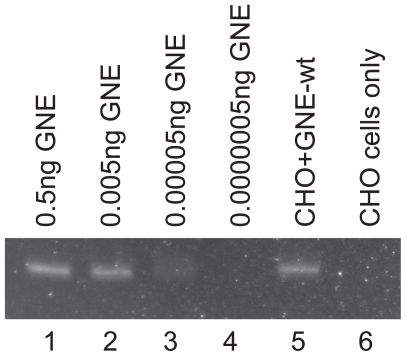
GNE mRNA is expressed in transfected CHO-Lec3 cells, but not in control cells. Lanes 1–4 contain 15 ul of serial diluted pUMVC3-GNE-wt PCR product, which was used to quantitate the amount of GNE mRNA present in the Lec3 samples. Lanes 5–6 contain 15 ul of the PCR product from transfected or untransfected Lec3 cells.

CHO-Lec3 cells were grown in 10% serum and transiently transfected with pUMVC3-GNE-wt DNA for 24 h to quantitate the amount of recombinant GNE RNA was expressed. Total RNA was extracted and RT- QPCR was performed to amplify a 230 bp fragment from the GNE transcript. Serial dilutions of pUMVC3-GNE-wt were used to determine that the concentration of GNE-wt expressed in transfected Lec3 cells was equal to 4.1 pg/ul. The dynamic range of the QPCR was from 5ng–5fg and there was no GNE mRNA product detected in control (untransfected) CHO-Lec3 cells (the cT value for untransfected cells was greater than 42 cycles, which is less than 5fg). Therefore, we were able to detect recombinant GNE mRNA expression in transfected Lec3 cells, while untransfected cells had undetectable amounts of GNE mRNA. (Fig. 4).

### GNE enzyme assays

In addition to the western blot assay, an aliquot of the transfected cell pellets were assayed for enzyme activity. Both epimerase and kinase activity were quantified in Lec3 cells with or without recombinant GNE protein (Table 1). Lec3 cells alone had both epimerase and kinase activities less than 3 mU/mg, which displays background activity. Cells expressing wild type, M712T, or R266Q GNE had an average of 22, 31, and 26 mU/mg of epimerase activity, respectively. The same Lec3 samples displayed an average of 35, 32, and 38 mU/mg of kinase activity. All of the cells expressing recombinant GNE had enzyme activity significantly above the non-treated cells with a p-value ≤ 0.008 for both epimerase and kinase activities. Using one-way ANOVA, followed by Duncan’s multiple comparison, there was no statistical difference in enzyme activity between the three different GNE isoforms, with p-values greater than 0.05.

### Sialic acid assays

Transfected Lec3 cells also were tested for cell surface sialic acid expression. All Lec3 samples had approximately 6.0 nmol/mg membrane bound sialic acid, with the exception of Lec3 cells transfected with the R266Q GNE, which had a 1.5-fold higher amount. The R266Q mutant lacks the feedback inhibition of GNE and is known to cause an overproduction of intracellular sialic acids. Lec3 cells seem to be undersialylated, and this could only be overcome by expression of the sialuria mutant and not by the about 100-fold overexpression of wild-type GNE compared to wild-type CHO cells.

No differences between wt and M712T GNE were observed. This was likely due to the incorporation of sialic acids from the cell culture medium, as it is known that sialic acids from FBS can bypass the defective GNE pathway (Hong and Stanley, 2003; Hinderlich, Salama et al. 2004). In this case differences between wt and M712T could be masked by the bypass. We therefore altered the cell culture conditions by reducing the percent serum (FBS) in the media. As the serum level was reduced, sialic acid production decreased, with a marked decrease demonstrated at 2.5% FBS (Table 2). Sialic acid levels continued to decrease as the cell culture media approached serum free conditions, but the cell morphology and growth characteristics were altered (data not shown). It was determined that the 2.5% FBS concentration of the cell culture media was optimal in order to test the impact of GNE gene transfection in Lec3 cells.

**Figure 5:**
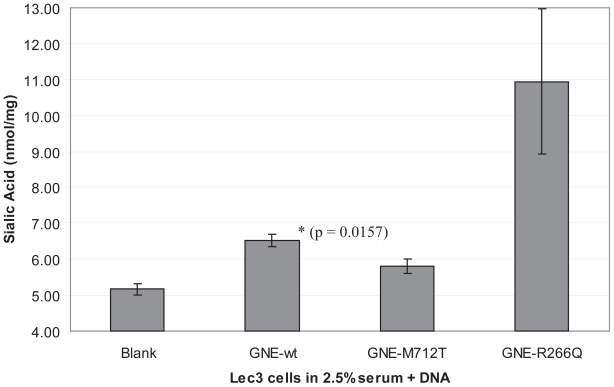
Sialic acid is reconstituting by GNE expression in CHO-Lec3 cells cultivated in the presence of 2.5% FBS. In comparison to untreated Lec3 cells, sialic acid production was significant greater following GNE-wt (p = 0.0157) transfection. GNE-R266Q (p = 0.0566) and GNE-M712T (p = 0.0708) approached significance.

Lec3 cells were thus grown in 2.5% FBS and transfected with pUMVC3-GNE clones. GNE expression was concurrently confirmed via western blot (Fig. 3). Significant increase of sialic acid production was indeed demonstrated, again with the best effect of the R226Q mutant (Fig. 5, p = 0.0157 for GNE-wt; p = 0.0566 for GNE R266Q). A slight, but significant difference between wt and M712T GNE was observed, indicating, that the re-sialylation capability of the mutant is lower than that of the wild-type, likely hinting at a similar mechanism in HIBM muscle.

## Discussion

We created several GNE expression vectors from human cDNA. Three different GNE forms, wild type, M712T, and R266Q, were robustly expressed in GNE deficient cells (Lec3 cells). All enzymes demonstrated similar protein expression levels, albeit distinct enzymatic activities. The transfected GNE expressing cell lines produced significantly more sialic acid than untransfected cells.

Studies on HIBM2 reveal mutations in the GNE gene associated with glycosylation errors in the muscle membrane which may lead to defective muscle function. Loss of GNE activity in HIBM2 is thought to impair sialic acid production and interfere with proper sialylation of glycoconjugates (Huizing, Rakocevic et al. 2004; Saito, Tomimitsu et al. 2004). The reactivities to lectins are also variable in some myofibers, suggesting that hyposialylation and abnormal glycosylation in muscles may contribute to the focal accumulations of autophagic vacuoles and/or amyloid deposits in affected patient muscle tissue.

Sialic acids can be found as part of cell surface glycoproteins, glycolipids, gangliosides, and polysaccharides and form a large part of the glycocalyx which plays a large role in the function of the cell membrane. The activity of the enzyme necessary for sialic acid production can be controlled at the transcriptional level (Oetke, Hinderlich et al. 2003). The negative charge of the cell surface is ascribed to sialic acid with an affinity for various cations, particularly calcium (Ca^++^). The glycocalyx on the cell surface blends with the ground substance of the interstitial space and abuts the basement membrane of closely applied capillaries (Frank and Langer, 1974). This relationship provides for a significant amount of capillary-cellular exchange. Experimental observation reveals a significant variability in muscle contractile force based on Ca^++^ availably, when artificially altered in the vascular supply (Shine, Serena et al. 1971). The bound Ca^++^ in the glycocalyx appears to be in rapid equilibrium with Ca^++^ in the vascular and interstitial spaces and is the probable immediate source of the Ca^++^ that crosses the sarcolemma. The Ca^++^ bound at the surface also seems to be important in the excitation-contraction (EC) coupling sequence, whether as a source of “trigger” Ca^++^ for the sarcotubular system or as a direct activator of the myofilaments. The integrity of the sialic acid component of the glycocalyx appears to be necessary for the binding of Ca^++^ and for the prevention of uncontrolled Ca^++^ entry into the cell allowing for effective muscle contraction (Langer, 1978).

Attempts to palliate HIBM2 progression with Sialic Acid, ManNAc or saliated protein replacement, which bypass the defective epimerase and/or kinase related to the GNE mutation, are being explored in various experimental trials. Supportive preliminary preclinical results suggest potential improvement in function of GNE mutated fibroblasts (Huizing, Hermos et al. 2002). A novel homozygous mutant (GNE^M712T/M712T^) mouse model which does not develop myopathy but does develop early renal failure and death related to kidney dysfunction was shown to have transient improvement in survival following treatment with ManNA protein. These mice unfortunately do not demonstrate the functional myopathic abnormalities associated with HIBM2. HIBM2 patients do not have associated renal dysfunction, and thus this model is of limited use for evaluation of clinical product testing (Galeano, Klootwijk et al. 2007; Quaggin, 2007). Some groups have tried to create a GNE deficient mouse model containing myopathic related characteristics, but the homozygous mutants die in utero (Schwarzkopf, Knobeloch et al. 2002; Malicdan, Noguchi et al. 2007). However, the M712T mouse model may help point to possible therapies which ultimately will require rigorous clinical validation. GNE-null mice bearing the D176 GNE human transgene do show myopathy reminiscent of HIBM2 after 10 months of age (Malicdan, Noguchi et al. 2007), however the molecular basis of myopathy in this model remains controversial since the muscle GNE expression is higher than expected physiologic levels. Additionally, due to multi-organ problems and unpredictable survival, the utility of this mouse model for pre-clinical trials remain suboptimal. We investigated the effect of a novel GNE gene/CMV promoter plasmid for mRNA and protein expression in GNE deficient CHO-Lec 3 cells and were able to restore GNE/MNK enzyme function and subsequent induction of sialic acid production.

Preclinical studies with myogenic stem cells have failed to show functional improvement in muscle function (Partridge, Morgan et al. 1989; Barr and Leiden, 1991; Dhawan, Pan et al. 1991; Mendell, Kissel et al. 1995). Development of a relevant mouse model would facilitate the pre-clinical evaluation of potential therapeutic approaches to HIBM2. However, as no such model exists, clinical testing in advanced patients with products of sound scientific rationale with supportive *in vitro* evidence of efficacy along with *in vivo* demonstration of product safety, is justified.

It has been documented that metabolic precursors in serum can be taken up by cells in culture and converted to glycoconjugate-bound sialic acids (Oetke, Hinderlich et al. 2001; Hinderlich, Salama et al. 2004). In the data presented here, we demonstrated that Lec3 cells grown in 2.5% serum had significantly less sialic acid expression than cells grown in normal (10%) serum conditions. We were able to culture the cells in serum free media and obtain sialic acid levels close to 0.0 nmol/mg, but the cell morphology and growth characteristics were noticeably different from the original cells. Therefore, cells in 2.5% serum were used for sialic acid reconstitution assays, but the minimal amount of serum present may have prevented us from displaying differences between the wild type and M712T GNE forms more clearly.

The expression vector in this study utilizes the robust CMV promoter. This results in relatively high levels of GNE protein expression. Therefore, few copies of plasmid DNA are required to be transfected into a cell to obtain physiological effects. In addition, the wild type GNE enzyme will be subject to normal feedback regulation precluding overexpression of sialic acid. Although the vector will not integrate into the chromatin, extended expression is expected in muscle tissue because myocytes do not rapidly divide. One could also consider use of the sialuria variant of GNE for expression in HIBM patient’s cells to rescue the long-time hyposialylation of the tissue, of course taking into account the side effects of sialic acid overproduction observed in sialuria patients. We are currently validating a non immunogenic muscle targeting delivery vehicle to determine the safety of the pUMVC3-GNE-wt vector in animals in preparation for clinical testing.

## Supplementary Material

**Figure S1:**
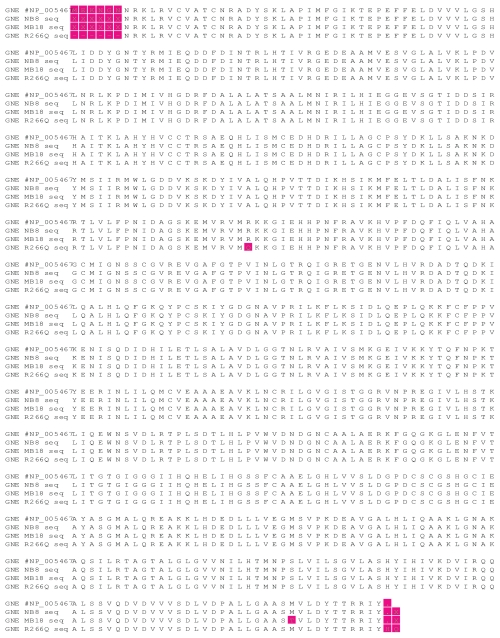
Sequence alignment of GNE wt (NB8), M712T (MB18), and R266Q (R266Q). Original DNA sequence was converted to protein to illustrate the amino acid mutations.

## Figures and Tables

**Table 1:** GNE enzyme activity of CHO Lec3 cells transfected with different plasmids.

Lec3 cells+DNA	Epimerase act (mU/mg)	p-value	Kinase act (mU/mg)	p-value
Untreated	1 ± 0.7		2 ± 1.4	
WT GNE	22 ± 0.6	0.001*	35 ± 0.7	0.001*
M712T GNE	31 ± 1.4	0.001*	32 ± 2.1	0.003*
R266Q GNE	26 ± 2.9	0.007*	38 ± 4.2	0.008*

* comparison to untreated.

**Table 2:** Sialic acid levels of CHO Lec3 cells cultivated in different concentrations of FBS.

% FBS	Sialic acid (nmol/mg)	p-value
10	8.05 ± 0.27	
5.0	7.26 ± 0.61	0.2996*
2.5	4.69 ± 1.20	0.0096*

* comparison to 10% FBS.
